# IN THE EYES OF THE BEHOLDER: RACE, PLACE, AND HEALTH

**DOI:** 10.1093/geroni/igac059.877

**Published:** 2022-12-20

**Authors:** Alfredo Velasquez, Jason Douglas, Fangqi Guo, Jennifer Robinette

**Affiliations:** Chapman University, Orange, California, United States; Chapman University, Orange, California, United States; Chapman University, Chapman/Orange, California, United States; Chapman University, Chapman/Orange, California, United States

## Abstract

Racial and ethnic health disparities are fundamentally connected to neighborhood quality. For example, racial and ethnic minorities are more likely to live in neighborhoods with signs of physical disorder (e.g., graffiti, vandalism), and physically disordered environments have been noted to associate with increased risk for chronic illness. Given that older adults may spend more time in their neighborhoods than younger adults as they transition out of the workforce, examining associations between neighborhood physical disorder and health among older minorities is of critical importance. Using 2016-2018 Health and Retirement Study (HRS) data, a representative sample of US adults aged 51 years and older (n= 9,080, mean age 68 years), we conducted a series of weighted linear regressions to examine links between neighborhood disorder as rated by third parties and both participant-perceived neighborhood safety and self-rated health. Study results indicated that higher neighborhood physical disorder was significantly related to more neighborhood safety concerns among non-Hispanic White and Hispanic residents, but not among non-Hispanic Blacks. On the other hand, neighborhood physical disorder was significantly associated with poorer health among all racial/ethnic groups. These patterns persisted after adjusting for education, sex, age, and census tract concentrated disadvantaged, population density, and racial/ethnic diversity. Our results indicate that community level interventions targeting neighborhood physical disorder may improve community health and minimize racial/ethnic health disparities.

